# The Quest for Outpatient Mastectomy in COVID-19 Era: Barriers and Facilitators

**DOI:** 10.1155/2022/1863519

**Published:** 2022-06-01

**Authors:** L. J. van Zeelst, R. Derksen, C. H. W. Wijers, J. Hegeman, R. Berry, J. H. W. de Wilt, L. J. A. Strobbe

**Affiliations:** ^1^Canisius Wilhelmina Hospital, Department of Surgical Oncology, Nijmegen, Netherlands; ^2^Canisius Wilhelmina Hospital, Department of Anaesthesiology, Nijmegen, Netherlands; ^3^Canisius Wilhelmina Hospital, CWZ Academy, Nijmegen, Netherlands; ^4^Radboudumc, Department of Surgical Oncology, Nijmegen, Netherlands

## Abstract

**Background:**

The rate of inpatient mastectomies remains high despite multiple studies reporting favourably on outpatient mastectomies. Outpatient mastectomies do not compromise quality of patient care and are more efficient than inpatient care. The objective of this study was to evaluate the feasibility of outpatient mastectomy.

**Materials and Methods:**

Implementation of an outpatient mastectomy program was evaluated in a retrospective study. All patients who underwent mastectomy between January 2019 and September 2021 were included.

**Results:**

213 patients were enrolled in the study: 62.4% (*n* = 133) outpatient mastectomies versus 37.6% (*n* = 80) inpatient mastectomies. A steady rise in outpatient mastectomies was observed over time. The second quarter of 2020, coinciding with the first COVID-19 wave, showed a peak in outpatient mastectomies. The only significant barrier to outpatient mastectomy proved to be bilateral mastectomy. Unplanned return to care was observed in 27.8% of the outpatient versus 36.3% of the inpatient mastectomies (*P*=0.198); the reason for unplanned return of care was similar in both groups.

**Conclusions:**

Outpatient mastectomy is shown to be feasible and safe with a steady increase during the study period. A barrier to outpatient mastectomy was bilateral mastectomy. Incidence of unplanned return to care or complications did not differ significantly between the outpatient and inpatient cohorts.

## 1. Introduction

For over twenty years, studies report outpatient breast cancer surgery to be feasible and safe [1, 2]. However, rates of inpatient mastectomy remain high worldwide and in the Netherlands, with most patients admitted for 1-2 nights [3–6]. The idea that outpatient mastectomy is unsafe and not patient friendly remains the primary concern.

Earlier studies reported no decrease in quality of patient care in patients receiving outpatient mastectomy. It does not affect postoperative complications or return to care, consisting of additional visits to the outpatient clinic or emergency department (ED), readmissions, and reoperations in comparison with inpatient mastectomies [1–3, 7–10]. Advantages of outpatient treatment have been reported; recovery in a familiar environment improves patient satisfaction [3]. Depression and anxiety rates are reported lower and patient control and independence rates are increased [2, 11, 12]. Physical recovery is improved in patients with an early discharge due to a decrease in postoperative pain and shoulder function impairment [8, 11]. Moreover, early discharge reduces the risk for nosocomial infections and delirium in the elderly [2, 13]. In addition to improved patient comfort, outpatient mastectomy results in more efficient care, since healthcare consumption is reduced and clinical resources can be used for higher acuity patients [3, 12]. Unfortunately, geographical differences in reimbursement policies sometimes encourage longer stay because of budgetary incentives.

To treat mastectomy patients safely in outpatient care, a multifactorial design is needed [1, 8]. In the Canisius Wilhelmina Hospital (CWZ), a mastectomy program was initiated to increase outpatient treatment and home recovery following mastectomy. We hypothesized outpatient mastectomy using the quilting technique and drainless surgery, as an adjunct to comprehensive pre and postoperative pathways without compromising quality of patient care which is feasible for a majority of patients. In a retrospective study, we evaluated the implementation of the mastectomy program; the main goal was to evaluate feasibility of outpatient mastectomy. Facilitators and barriers to outpatient mastectomy were identified in order to improve compliance.

## 2. Methods

### 2.1. Study Design

A retrospective cohort study was conducted in CWZ in Nijmegen, the Netherlands, from January 2019 to September 2021. CWZ provides breast cancer care to approximately 330 newly diagnosed patients annually. The local Ethical Committee of CWZ approved the study, complying with current regulations. Informed consent was not required because of the retrospective nature of the study.

### 2.2. Participants

All patients of 18 years and older undergoing mastectomy in CWZ, including those undergoing bilateral mastectomies and mastectomy with axillary lymph node dissection (ALND), were included in the study. Patients receiving direct breast reconstruction, including prosthetic and autologous flap reconstruction, were excluded, since standard practice includes drains and overnight stay.

### 2.3. Procedure

A mastectomy program was developed and gradually implemented in CWZ in 2019; the entire surgical-anaesthetic team was included. The main goal was to enhance perioperative care and improve outpatient treatment. Correct and ensuring information was given to patients from initial consultation. Patients were educated about the normal postoperative course and wound care by breast surgeons and breast care nurses to create confidence. Concerns regarding pain, wound management, and the reassurance of self-dependency following mastectomy which, albeit associated with considerable psychological impact, have limited impact on one's physical abilities were discussed. It was emphasized to contact the hospital if there were any uncertainties. Contact information (phone, e-mail) was provided during the first consultation. Patients were educated about the decreased risk of nosocomial infections, deep venous thrombosis, and delirium in the elderly when treated in outpatient care. A protocol regarding perioperative pain management was designed. The main goal was to get patients more physically fit out of surgery. In order to achieve this, the use of morphine and gas-based anaesthetics was minimized, while propofol and remifentanil were administered as anaesthetics. Perioperative patients received dexamethasone and ondansetron to reduce postoperative nausea. To achieve standardized anaesthetics in mastectomies, anaesthesiologists were given supporting materials, consisting of multiple presentations and standardized order sets. A perioperative serratus and interpectoral block with ropivacaine 0.2% 20–40 ml was performed to reduce postoperative pain. The use of antibiotic prophylaxis (1 g cefazolin 1 hour before incision) was limited to patients undergoing ALND. In addition, prior to subcutaneous and skin closure, skin flaps were sutured to the pectoral muscle using the quilting suture technique as described elsewhere [14]. Postoperative drains were omitted. All patients received postoperative compressive dressing or a customized bra. They were encouraged to mobilize the shoulder postoperatively. The breast care nurse telephoned the patient on the first postoperative day. The first postoperative visit to the outpatient clinic was planned two weeks after surgery.

### 2.4. Data Collection and Definition

Patient characteristics including age, BMI, ASA classification (as a proxy for comorbidity), polypharmacy, smoking status, and type of surgery (including uni and bilateral mastectomy or mastectomy with ALND) were obtained.

Primary outcome was the rate of outpatient treatment following mastectomy: outpatient treatment was defined as same calendar day discharge; if there was at least one overnight stay, it was defined as inpatient admission. Secondary outcomes were compared between outpatients and inpatients. Secondary parameters were reason for unplanned inpatient admission, return to care within 30 days postoperative including unplanned postoperative visits to the outpatient clinic or ED, readmission, and reoperation, and the reasons therefore include wound complications (seroma, surgical site infection (SSI), bleeding complications, and wound healing problems), infections not localized to the surgical site, venous thromboembolism, wound concerns, and wound pain [15].

### 2.5. Statistical Analysis

SPSS (IBM SPSS statistics for Windows, Version 26.0) was used to analyse data. Summary statistics were calculated for patient, clinical, and surgical characteristics. Normally distributed continuous data were presented as mean ± SD, and the independent *t*-test was used to analyse data. Categorical data were presented as numbers with percentages and were analysed by the chi square test or Fisher' exact test. Univariable logistic regression analyses were performed to identify predictors for outpatient treatment. Multivariable logistic regression analysis were not performed due to small amount of patients in the subgroups. *P* value < 0.05 was considered statistically significant.

## 3. Results

**Figure 1:**
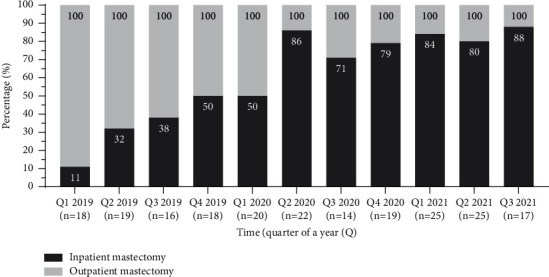
The evolution of outpatient mastectomy over more than two and half a year, stabilizing around 80% in 2021.

A total of 213 patients were enrolled in the study: 62.4% (133) outpatient mastectomies versus 37.6% (80) inpatient mastectomies. A steady rise in outpatient mastectomies was seen over time. The second quarter of 2020 showed a peak in outpatient mastectomies (Figure 1), coinciding with the first COVID-19 wave.

Cohorts of outpatient and inpatient mastectomies were compared. Patient characteristics were not significantly different regarding age, BMI, smoking status, polypharmacy, and ASA classification. The type of surgery was significantly different, with unilateral mastectomy more often performed in the outpatient cohort and bilateral mastectomy and mastectomy with ALND more often performed in the inpatient cohort (Table 1).

19 patients were planned as outpatient mastectomies but converted postoperatively to inpatient mastectomies. Most common reasons were delayed recovery due to malaise (6 patients, 31.6%), pain (3 patients, 15.8%), and nausea (5 patients, 26.3%). One patient was admitted because observation of postoperative hematoma was needed, and one patient was admitted because surgery finished late. Reason for inpatient admission was unknown in three patients.

Unplanned return to care was observed in 37 patients (27.8%) of the outpatient versus 29 (36.3%) of the inpatient cohort (*P*=0.198). The number of additional visits was not significantly different between the cohorts. Reasons for return to care did not differ significantly between the cohorts; the most common reasons for return to care were seroma, bleeding complications, wound concerns, and pain. In the outpatient cohort, one patient was readmitted and reoperated because of a bleeding complication versus none in the inpatient cohort (Table 2). Bilateral mastectomy emerges as a barrier to outpatient treatment with an OR of 13.33 (95% CI 1.61–110.79, *P*=0.016).

## 4. Discussion

The implementation of a multifactorial mastectomy program resulted in a steady increase of outpatient mastectomies over more than two years and a half in CWZ. A boost in outpatient procedures was observed during the first lockdown of the COVID-19 pandemic, followed by gradual adaptation and stabilization at >80% outpatient procedures in 2021. Outpatient mastectomies in bilateral mastectomy lags behind in this study. Unplanned return to hospital care due to complications did not differ.

Since the introduction of the outpatient mastectomy program in CWZ in 2019, a gradual increase in patients undergoing outpatient mastectomy was observed (Figure 1). This gradual increase was partially explained by caregivers who had to become accustomed to outpatient treatment in order to convey certainty and trust to the patients. In addition, patients expecting overnight stay, for decades having seen relatives and acquaintances as inpatients, were sometimes reluctant. In 2019, 32.4% of mastectomies were outpatients, and in 2020, this increased to 72% and to 83.6% in the first three quarters of 2021. This is in line with earlier reported outcomes: 23% as outpatient mastectomies before implementing a mastectomy program, compared to 61% after implementation [2]. The first lockdown of the COVID-19 pandemic (March 2020 until June 2020) gave an extra boost to outpatient treatment peaking at 86%. During the COVID-19 pandemic, patients were discharged as soon as possible in order to limit the use of facilities, compulsory reserved for COVID-19 patients. Since the urge for early discharge was high during the first lockdown of the COVID-19 pandemic, we might conclude that 90% outpatient treatment seems a realistic aim.

Baseline characteristics between the cohorts were comparable except for the type of surgery. Bilateral mastectomy was mainly an inpatient treatment: 8 patients underwent bilateral mastectomy of whom only 1 (12.5%) had an outpatient mastectomy. This is in line with earlier studies reporting 35% of bilateral mastectomies as outpatients resulting in lower odds of outpatient treatment (OR 0.70; 95% CI 0.54–0.91) [2, 7]. All but one patient undergoing bilateral mastectomy were planned as inpatients before surgery. The most likely explanation is that caregivers feel they ought to offer some extra facilities to patients harder hit by breast cancer. A lesson learned is to motivate all involved professionals for a change in procedure with the appropriate evidence. However hard the psychological impact of a bilateral mastectomy may be, the general anaesthesia and the associated recovery is comparable to that of a unilateral operation. In earlier studies, comorbidity (presented as a higher ASA classification) was reported as a predictor for failure of outpatient mastectomy [1, 7]. In our study, the difference in ASA classification was not significant nor clinically relevant.

Intractable vomiting, patient anxiety, and pain control were reported to be main reasons for failing discharge [3]. In this study, the main reasons for failing discharge were malaise, pain, and nausea. It is debatable whether these reasons necessitate an inpatient admission. A few decades ago, patients were admitted to the hospital for a week after chemotherapy in order to deal with complaints as malaise, nausea, and pain. Nowadays, patients receiving chemotherapy are discharged the same day, despite the fact that most of these patients will experience malaise, pain, and nausea. Problems as malaise, nausea, and pain can be anticipated on through directed preoperative information, reassurance, and appropriate medication. A surgeon' visit at the end of the day may help in alleviating anxiety and associated complaints.

Unplanned return to care was higher in inpatient mastectomies: 36.3% in inpatient mastectomies versus 27.8% in outpatient mastectomies. This difference, however, not statistically significant, might be of clinical relevance. Earlier studies reported no significant differences in unplanned return to care between outpatient and inpatient mastectomies [2, 8]. Patients staying one night had higher odds of postoperative complications (OR 1.37; 95% CI 1.16–1.63, *P*=0.004), and patients admitted to the hospital for more than one night had over twice the odds of postoperative complications (OR 2.65, 95% CI 2.21–3.18, *P* < 0.0001) compared to patients treated in outpatient care. Despite a multivariable analysis to adjust for confounders, the postoperative length of stay remained a significant predictor for complications [1]. Other studies reported African American patients or patients with higher ASA classification to be more likely to return for unplanned care [1, 7]. In the present study, ASA was not found to be a barrier to outpatient surgery. Neither the amount of unplanned visits nor the reasons for return to care were significantly different between the cohorts. In both groups, the main reasons for return to care were related to seroma, bleeding complications, postoperative pain, and wound concerns. This is comparable to the literature, in which wound checks, concern for bleeding, and drain concern were the most common reasons for patients to return to care [7]. In this study, there was only one single patient who was reoperated and readmitted to the hospital because of postoperative hematoma. Earlier studies reported no statistically significant differences in reoperation or readmission rates [2]. Reasons for readmission included surgical site complications, infection not localized to the surgical site, and venous thromboembolism [15].

Patients receiving immediate breast reconstruction were excluded. In CWZ, standard practice following prosthetic reconstruction includes drains and overnight stay. Postoperative drainage is a known barrier to outpatient treatment in literature and in our hospital [5]. Also, prosthetic retropectoral reconstruction, being the plastic surgeons preferred technique, is more painful than simple mastectomy. Outpatient treatment is not possible for patients receiving autologous breast reconstruction, since the skin island perfusion has to be monitored. Direct reconstruction was reported to be a significant barrier to outpatient treatment [7]. However, in multiple studies, outpatient treatment of mastectomy with direct reconstruction is concluded feasible without increasing postoperative complications, readmissions, or ED visits [16, 17].

Outpatient mastectomy results in more efficient care. The average costs in CWZ are calculated at *€*5.491 for outpatient mastectomy versus *€*7.486 for inpatient mastectomy. In the Netherlands, 15000 women are diagnosed with breast cancer, and one out of three undergo mastectomy, resulting in 5000 mastectomies per year [18, 19]. In the optimal situation, 90% of these are being treated as outpatient, and this could result in an annual estimated cost reduction of *€*8.977.500.

Strengths of this study are its real life design demonstrating that a dedicated team effort can result in a successful outpatient mastectomy program. Complete data from electronic patient files were gathered over a period of two and half a year, making it possible to present a reliable timeline of the evolution to outpatient mastectomy. However, there are some drawbacks relating to the retrospective design of the study as hidden confounders cannot be ruled out. The limited study population can preclude some trends to become significant. The single centre design implicates geographical and cultural (reimbursement) confounders.

## 5. Conclusion

Feasibility and safety of outpatient mastectomy have been confirmed in this study. A comprehensive protocol, including the entire surgical-anaesthetic team, is of paramount importance. A steady increase in outpatient mastectomies was observed over two and half a year, stabilizing around 80% in 2021, with the COVID-19 pandemic proving to be a leverage. Bilateral mastectomy seems to be the most important barrier to outpatient mastectomy. Unplanned return to care did not differ between outpatient and inpatient treatment.

## Figures and Tables

**Table 1:** Patient and baseline characteristics.

	Outpatient mastectomy, *n* = 133 (%)	Inpatient mastectomy, *n* = 80 (%)	*P* value
Age	63.4 ± 12.8	65.7 ± 14.7	0.440

BMI	26.3 ± 5.3	26.9 ± 4.8	0.180

Smoking status			0.534
Yes	16 (12.0)	12 (15.0)	
No	117 (88.0)	68 (85.0)	

Polypharmacy			0.377
Yes	102 (76.7)	57 (71.3)	
No	31 (23.3)	23 (28.7)	

ASA classification			0.068
I	52 (39.1)	20 (25.0)	
II	69 (51.9)	46 (57.5)	
III	12 (9.0)	13 (16.3)	
IV	0 (0)	1 (1.3)	

Type of surgery			0.007
Mastectomy unilateral	120 (90.2)	63 (78.8)	
Mastectomy bilateral	1 (0.8)	7 (8.8)	
Mastectomy with ALND	12 (9.0)	10 (12.5)	

Continuous variables are presented as mean ± standard deviation, and categorical variables are presented as frequency (%). BMI, body mass index; ASA, American Society of Anaesthesiologists classification; ALND, axillary lymph node dissection.

**Table 2:** Outcomes regarding return to care.

	Outpatient mastectomy, *n* = 133 (%)	Inpatient mastectomy, *n* = 80 (%)	*P* value
Unplanned RTC			0.198
Yes	37 (27.8)	29 (36.3)	
No	96 (72.2)	51 (63.7)	

Additional visits			0.435
0	96 (72.2)	51 (63.7)	
1	24 (18.0)	16 (20.0)	
2-3	12 (9.0)	11 (13.8)	
≥4	1 (0.8)	2 (2.5)	

Reason for RTC^*∗*^			0.694
CSS	12 (9.0)	7 (8.8)	
Nonaspirated seroma	5 (4.3)	2 (2.6)	
SSI	4 (3.0)	4 (5.0)	
Bleeding complication	7 (5.3)	6 (7.5)	
Wound healing problem	5 (3.8)	3 (3.8)	
Wound concerns	3 (2.3)	7 (7.5)	
Thromboembolic complication	1 (0.8)	0 (0)	
Pain	4 (3.0)	6 (7.5)	
Other reasons	1 (0.8)	0 (0)	

Readmission	1 (0.8)	0 (0)	0.372

Reoperation	1 (0.8)	0 (0)	0.372

Categorical variables are presented as frequency (%). RTC, return to care; CSS, clinical significant seroma; SSI, surgical site infection. ^*∗*^The sum of the reasons for RTC is > the amount of patients returning to care since several patients had ≥1 reason to RTC.

## Data Availability

The dataset generated during the current study is available from the corresponding author upon reasonable request.
