# Corrigendum to “The Quest for Outpatient Mastectomy in COVID-19 Era: Barriers and Facilitators”

**DOI:** 10.1155/tbj/9847254

**Published:** 2025-09-15

L. J. van Zeelst, R. Derksen, C. H. W. Wijers, et al., “The Quest for Outpatient Mastectomy in COVID-19 Era: Barriers and Facilitators,” *The Breast Journal* 2022 (2022): 1863519, https://doi.org/10.1155/2022/1863519.

In the article titled “The Quest for Outpatient Mastectomy in COVID-19 Era: Barriers and Facilitators” there were errors in Figure 1 and Table 1.

In Figure 1 legends, inpatient mastectomy and outpatient mastectomy were attributed to the wrong colour. The corrected Figure 1 is shown below.

In Table 1, values in the polypharmacy rows were incorrect. The corrected Table 1 is shown below.

We apologize for this error.

## Figures and Tables

**Figure 1:**
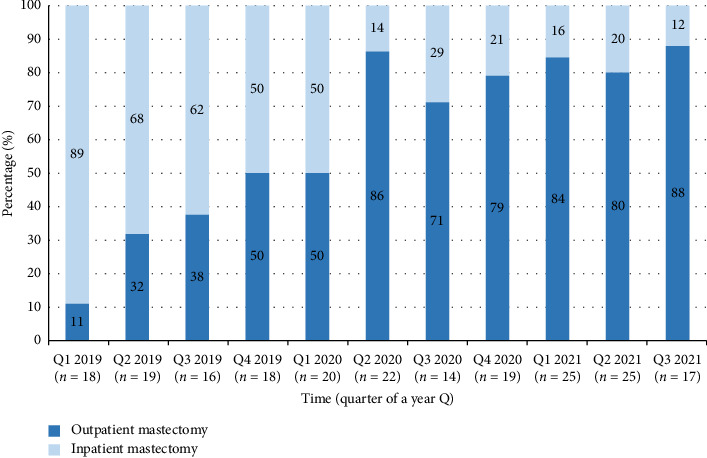
The evolution of outpatient mastectomy over more than two and half a year, stabilizing around 80% in 2021.

**Table 1:** Patient and baseline characteristics.

	Outpatient mastectomy *n* = 133 (%)	Inpatient mastectomy *n* = 80 (%)	*p* value
Age	63.4 ± 12.8	65.7 ± 14.7	0.440
BMI	26.3 ± 5.3	26.9 ± 4.8	0.180
Smoking status			0.534
Yes	16 (12.0)	12 (15.0)	
No	117 (88.0)	68 (85.0)	
Polypharmacy			0.377
Yes	31 (23.3)	23 (28.7)	
No	102 (76.7)	57 (71.3)	
ASA classification			0.068
I	52 (39.1)	20 (25.0)	
II	69 (51.9)	46 (57.5)	
III	12 (9.0)	13 (16.3)	
IV	0 (0)	1 (1.3)	
Type of surgery			0.007
Mastectomy unilateral	120 (90.2)	63 (78.8)	
Mastectomy bilateral	1 (0.8)	7 (8.8)	
Mastectomy with ALND	12 (9.0)	10 (12.5)	

*Note:* Continuous variables are presented as mean ± standard deviation, and categorical variables are presented as frequency (%). ASA, American, Society of Anaesthesiologists Classification.

Abbreviations: ALND, axillary lymph node dissection; BMI, body mass index.

